# Global infant formula: monitoring and regulating the impacts to protect human health

**DOI:** 10.1186/s13006-014-0020-7

**Published:** 2015-02-23

**Authors:** George Kent

**Affiliations:** Department of Political Science, University of Hawai’i, Honolulu, HI 96825 USA

**Keywords:** Infant formula, Nutritional adequacy, Baby foods, Human rights, Food system

## Abstract

Worldwide promotion of infant formula and other commercial baby foods is leading to increased use of these products, raising concerns about their impact on the health of infants. These products are made and marketed through a global system that extends beyond the control of separate nations. As the industry is increasingly globalized, there is a growing need for guidance, monitoring, and regulation.

This study suggests a path toward achieving better control of infant formula and other baby foods to ensure that infants and young children everywhere are well nourished. The negotiation of a new Optional Protocol on Children’s Nutrition, to be linked to the most relevant human rights treaty, the Convention on the Rights of the Child, would bring the major issues relating to infant formula and other baby foods to the attention of the global community and all national governments.

In the distant past most food came from nearby sources with little processing. There was little governmental control of local food systems. Producers were connected to consumers. Short distances allowed consumers to be in contact with producers and hold them accountable for their actions. This is still the pattern in many low-income regions of the world.

That pattern changed radically over the last two centuries. In much of the world, food production, processing, and marketing have become industrialized. International food trade has become an important factor. Many people now have little contact with the primary producers or the processors of their food. Food used to be produced mainly to provide the sustenance needed for the local community, but the producers’ dominant motivation has become the production of their own wealth. National governments have taken an increasing role in shaping their national food systems, especially in high-income countries.

Safety and other issues relating to food are now addressed, more or less effectively, through regulation by national and sub-national governments. However, as the food system is increasingly globalized, there is a corresponding need for guidance, monitoring, and regulation at the global level. This is especially true for countries that have little capacity to control their own food systems, and are vulnerable to pressures from outside.

This study focuses on the need for global regulation of infant formula and other commercial baby foods. The argument for regulation of the infant formula industry has been made before [1], but the need for it has grown rapidly since then.

The analysis here unfolds through several phases.

The accelerating global promotion of the product and the economic motivations for it are described in the section on the *Globalization of Infant Formula*.

Since it plays an especially large role in this globalization process, *China* is examined in the following section.

The perspective of one of the major suppliers of infant formula, New Zealand, is then presented in the section on *An Infant Formula Value Chain*. Highlighting the economic interests of the producers, this value chain analysis is quite different from what would be obtained if the interests of consumers were emphasized.

The section on *Information for the Industry* shows that information about the global growth of the infant formula industry is not available to the general public. It also points out the industry’s apparent inattention to health concerns.

While a great deal of attention has been given to improving short-term safety in the use of infant formula, the issue of its nutritional adequacy has been ignored. This is addressed in the section on *Safety and Nutritional Adequacy*. Regulations should consider both.

Infants are fed in different ways with different products in different contexts. Standard infant formula and the many variations on it are better than some other methods of feeding and worse than others. The section on *Feeding Alternatives* highlights the importance of clarifying the feeding options under discussion when assessing the merits of any particular method of infant feeding.

The section on *Global Guidance* shows that a great deal has already been accomplished in achieving agreement on principles that should guide infant and young child feeding.

There is now a need to build on and strengthen that consensus, especially in the face of increasing pressures to deliver questionable foods. The closing section on *Prospects for Global Regulation* discusses approaches to addressing the issues at the global level.

## Globalization of infant formula

The growth of global networks of infant formula production is closely linked to the globalization of milk production. In the past, milk was valued as a fresh product, and consumed near to where it was produced, with little processing beyond pasteurization and homogenization. “Milk sheds” covering small areas often were based on cooperatives among farmers who knew each other. Now, however, milk production has been industrialized, with processors purchasing milk from local farmers, often on a contract basis, in very hierarchical systems [2]. Local small-scale farmers have limited influence in this system, and many have lost their farms altogether. A few dairy businesses have become global in scale. The largest dairy exporter in the world is Fonterra, based in New Zealand. Fonterra and other New Zealand firms are promoting industrial-style milk production in Brazil, Chile, Uruguay, the U. S. Midwest, and Hawai'i [3]. This globalized dairy industry supplies the basic milk powder for most infant formula.

Making and marketing infant formula and other baby foods has become a very profitable business, so the products are being promoted with great vigor, especially in emerging economies. Many people have increasing incomes, making them potential customers. The manufacturers are stepping up their production, often through joint ventures and other novel business arrangements. The dynamics of this process have been well documented in Indonesia and many other countries [4-6].

In the Asia-Pacific region the market for commercial baby food grew to US$14.7 billion in 2011 [7]. The rapid growth is expected to continue:

In 2013 the infant formula market is still growing rapidly with the development of markets like Asia, particularly China with a growth rate close to 20% [per year], Eastern Europe, and in a lesser extent Middle East and Latin America. The development of the market is linked with the economic growth of those countries and its corollary the growing number of working women [8] p. 3.

In the Middle East and North Africa, the baby food market has been growing at a compound annual growth rate of 11.2 per cent during 2007–2012. Infant formula is recognized as the primary driver of the entire baby food market in the region [9].

US$41 billion was spent on milk formula globally in 2013 [10]. Retail sales of infant formula in China alone are expected to reach US$27 billion by 2017 [11].

Investments in infant formula manufacturing are being made in low-income countries not only because of their emerging middle class, but also because of their lower costs of production. This is illustrated by Nestlé’s plan to build a new infant formula factory in Mexico based on the expectation that it will export about 40 percent of its products to Latin America and the Caribbean [12]. Locating these factories in low-income countries may also be attractive because they are not likely to be controlled by the national governments as rigorously as plants in high-income countries.

In at least one case the investment is from a low-income country into a high-income country. A Chinese firm, Synutra International, is investing US$125 million to build an infant formula manufacturing plant in France [13].

## China

The globalization of the infant formula market is largely due the sellers’ interest in the huge market for the product in China. Like many other countries, China has a growing middle class with money to spend. But China is different in important ways. The most obvious is its enormous size, in terms of both population — more than 1.3 billion people, and geography — almost 10 million square kilometers. China has had high levels of economic growth for many years. Demand for infant formula in China was expected to grow to more than US$14 billion in 2014 [14].”

One unique feature in China’s infant formula history helps to explain the growth of this market sector. In 2008 it was discovered that some infant formula produced in China was tainted by deliberate contamination with melamine [15,16]. Low-cost melamine was added to give the appearance that the formula had proper nutrient levels, but it did not. The result was that at least six children died, and many became ill. Two Chinese executives were sentenced to death because of their participation in the crime [15].

The harm to the economy continues. Chinese people have become wary of infant formula made by Chinese companies, and go to great lengths to import the product. They pay high prices, and the practice has stimulated a variety of illegal activities such as thefts and the smuggling of formula into China. The high demand for formula in China has resulted in significant depletion of supplies elsewhere [17].

The vigorous thrust of Fonterra and others to sell formula and other baby foods to China has been described as a “goldrush” [18]. Entrepreneurs in Australia and many other places are trying to get a share [19].

The continuing impact of the melamine scandal is demonstrated by an installation by Chinese dissident artist Ai Weiwei [20,21]. In museums in Hong Kong, Singapore, and the Philippines, he created floor maps of China stacked with cans of infant formula. The map highlights the preoccupation of China with infant formula. An art review said it “draws attention to the issue of milk safety and ensuing scarcity of supply in China resulting from the melamine scandal [21].” Unfortunately, the installation and the review said nothing about how increasing consumption of safe infant formula might have a negative effect on the well-being of Chinese children and their mothers, and the country as a whole.

## An infant formula value chain

The publicly available discussions of the vigorous promotion of infant formula suggest that it is motivated primarily by the high profits that are anticipated. There are no reports from any sources that would suggest that the primary motivation is to improve the health of the infants and their mothers.

The emphasis on the potential profits is well illustrated by the Coriolis report, *Infant Formula Value Chain* [22]. Coriolis describes itself as “a boutique management consulting firm that focuses on food, consumer packaged goods, retailing, and food service (p. 2).” The firm is based in Auckland, New Zealand and designed its analysis to serve the New Zealand infant formula industry.

The Coriolis report is candid as it acknowledges, “Infant formula is typically defined as ‘birth to six months’; the product is then renamed for a range of reasons (primarily to avoid regulation and restrictions on advertising) (p. 9)”.

The report describes the structure of global infant formula manufacturing, with the top five formula manufacturers (Nestlé, Danone, Mead Johnson, Abbott, and Heinz) accounting for 56% of the world market (p. 10). It says an emerging group of Chinese firms is challenging the current order, but that is misleading. Many of the Chinese firms are involved in joint ventures together with the dominant western manufacturers.

In recent years, there has been little growth in infant formula sales in high-income countries. The major growth is in low-income countries, or more precisely, in so-called “emerging economies” where there are growing sectors of middle- and high-income people. “China is driving world growth, growing +50% more than the rest of the world combined (p. 13).” This had led to large investments in the infant formula industry in New Zealand and elsewhere.

The value chain analyzed in this report examined the major phases of the industry such as procurement of ingredients, processing, packing, marketing, and shipping. The major roles are those of dairy farmers, dairy processors, manufacturers and marketers, retailers, and consumers (p. 33). A can of infant formula that retails for NZ$47.50 in 2012 yielded about NZ$13.25 for the retailer, NZ$25.58 for the manufacturer, NZ$1.91 for the dairy processor, NZ$2.69 for the dairy farmer, and smaller amounts for other participants in the value chain (p. 34). Each of them gets a substantial return on their assets, with the highest estimated rate of return, 29%, going to multinational infant formula manufacturers (p. 36). This helps to explain why New Zealanders are interested in having more of the manufacturing take place in New Zealand, rather than shipping milk powder to manufacturers in Asia (p. 62).

The report describes a typical Singaporean consumer of infant formula, a six month old, Ong, in a household with two working parents. The child is in day care, so the mother “can’t afford not to work. As a result Ong is fed infant formula (p. 37).” The family’s monthly expenditure on infant formula is more than half the typical family’s expenditure for food at home (p. 39).

The report acknowledges, “The major global multinationals put a large part of their selling effort into health practitioners rather than retailers . . .; they are doing this as it works to sell product (p. 41).”

This is the only point at which health is mentioned in the report. Its primary concern is the profitability to parties at various stages of the value chain, and not costs and benefits of various kinds to the final consumers or to their societies. The high economic benefits to the manufacturers explain why infant formula is a growing globalized industry.

In the type of value chain analysis offered by Coriolis, the impacts on households are not considered, presumably because it was prepared for manufacturers and marketers, the parties who draw the major economic benefits. A value chain analysis that was based on health and economic impacts on children and their families would yield different results. Analyses based on health concerns would have to consider the extent to which the promotion of infant formula would displace breastfeeding and as a result impact the health of both the children and their mothers. In addressing the impacts on household economics, it would assess the impact of increased use of infant formula on household budgets. It would be possible to undertake value chain analyses that take account of a broad variety of impacts on all the affected parties, but that has not been done.

## Information for the industry

While the general public knows little about this new wave of infant formula promotion, there are many private organizations devoted to keeping the business sector informed. The accessibility of this information is limited by its high cost.

The Transparency Market Research study mentioned earlier [7], 109 pages, can be purchased with a single user license for US$2,695. The UBIC website offers a 42-page section of its study on-line at no cost. The entire UBIC study can be ordered for €14,1990, or about US$20,000.

As of March 2014, Research and Markets offered a number of studies:

- China Goat Milk Infant Formula Industry Report 2014 — from US$2,200
- Baby Food & Formula Manufacturers (Global) – from US$3,995
- Global Baby Food and Formula Industry Forecast to 2016 — Asia: An emerging Market for Organic Baby Food — from US$665
- China Infant Formula Milk Powder Market Report, 2013–2017 — from US$1,850
- Baby Foods and Infant Formula — Global Strategic Business Report — from US$4,950
- Global and Chinese Infant Formula Milk Powder Industry Report 2014 — from US$2,200
- Research Report on Infant Formulas Market in China — from US$6,505
- Research Report on Infant & Young Child Formula Milk Powder Market in China 2012 — from US$6,600

Euromonitor International claims to have “the world’s most comprehensive research on the baby food category within the packaged food industry [23]. Their country-based studies sell for US$900 each. The prices on their studies of particular manufacturers vary, from $525 on up.

In June 2014 Technavio published a 68-page report, *Global Baby Food and Infant Formula Market 2014–2018*. A single user license for it is available for US$2500.

These reports offer estimates that cannot be verified easily, so their credibility may be questionable. Nevertheless, their prices suggest that purchasers anticipate good returns on their investments in them.

The summaries of these reports that are available to the public at no cost suggest that the business sector is giving little attention to the likely impact of the use of these products on health or on the economic status of families. Euromonitor discusses breastfeeding, but only to assess the impact of changing breastfeeding rates on the retail performance of baby foods, and to ask whether government initiatives to promote breastfeeding are constraining the market performance of baby foods. These reports are intended for the business sector, so it is not surprising that they give little attention to health impacts. The troubling thing is that no major agencies are monitoring the health impact of the huge growth in infant formula consumption.

Some data on baby food trade are available at no cost from global agencies such as the World Trade Organization and the Food and Agriculture Organization of the United Nations. However, their data are highly aggregated, and little is said about infant formula in particular. There is no way to know how much infant formula is traded internationally. Even if those data were available, they would not reveal the extent to which formula is promoted and supplied through international joint ventures. The international agencies do not provide data about specific corporate actors. Little information is offered partly due to the need to protect corporate proprietary interests, and also because the industry has little interest in opening itself to public scrutiny.

The United Nations Children’s Fund published a study on political commitments worldwide to protect, promote and support breastfeeding [24]. However, it did not assess the countervailing pressure, the steady reduction of breastfeeding resulting from the worldwide promotion of infant formula and other foods for infants and young children. This is not being monitored, and it is rarely discussed. This would not be a major concern if infant formula was simply a good business and it was not likely to have much impact on the health of infants or the children and adults they will become. However, as indicated in the following section, there is robust scientific evidence that feeding infants with formula rather than breastfeeding is likely to result in worse health for the children and also for their mothers. Some impacts, such as cognitive impairments, could last throughout their lives, and might even impact future generations.

## Safety and nutritional adequacy

The health risks associated with the use of infant formula were first brought to the world’s attention in 1939 when Dr. Cicely Williams spoke on *Milk and Murder*, railing against the dangers of using the product in low-income countries [25]. Challenges against infant formula rose to a high level in 1974 with the publication of *The Baby Killer* [26,27].

In the past, campaigns against the use of the infant formula focused on its frequently being used in unsafe ways, and on the risks of its contamination, especially in low-income countries. There have been significant improvements in the quality of infant formula, and a great deal has been done to ensure that it is prepared safely. Nevertheless, it remains true that many health outcomes are worse for formula-fed infants than for breastfed infants, including in high-income countries [28-32]. They are not as bad as the health outcomes in low-income countries, but they are bad and require attention. Apart from special conditions such as infants with certain disorders, at the population level every type of infant formula produces worse health outcomes than breastfeeding, no matter how carefully it is handled.

Many people have the impression that in high-income countries, where contamination and mishandling of infant formula is not a major issue, there is no problem with using the product. This is a serious error. There is a need to give attention not only to whether infant formula is safe, but also to whether it is effective in doing what it is supposed to do, its functionality. Saying that a food won’t make you sick right away is not the same as saying that it meets your needs. The studies cited in the preceding paragraph show that in all sorts of conditions, outcomes with formula feeding are consistently inferior to those obtained with breastfeeding. In some cases the outcomes may be only slightly inferior and in others greatly inferior, but I know of no credible studies that show formula feeding to be equal to or superior to breastfeeding in any general population. This is the pattern even when there are no safety issues. This means that infant formula is *nutritionally inadequate* when compared with breastfeeding. Of course, infant formula can play an important role in sub-populations with specific problems.

While the death and disease associated with unsafe use of infant formula usually shows up quickly, the harm to health that results from nutritional inadequacy usually is more subtle and slow to appear. A study that follows children only up to age seven [33], for example, will miss important long-term impacts, such as overweight, cognitive impairment, and susceptibility to various diseases throughout the lifespan. The failure of infant formula feeding to produce good results on these long-term impacts usually are not described as safety issues. This is why attention needs to be given to not only to the safety but also the nutritional adequacy of infant formula.

The common understanding is that food safety is about whether disease or death is likely to result from consuming the food product in the short term. This conforms with the United States government’s definition: “safe” means “a reasonable certainty in the minds of competent scientists that the substance is not harmful under the intended conditions of use”, as specified in the U.S. Code of Federal Regulations at 21 CFR 170.3(i). Often, concerns about safety are triggered by a record of “adverse reactions” to consumption of the product.

Food safety is about harms that are likely to make consumers worse off than they would have been if they had not consumed the food [34]. However, concerns about health benefits focus on whether consumers are better off as a result of consuming the food. When evaluating food products it is important to give attention not only to harms but also to benefits. Obviously, infant formula should do no immediate harm. It should also provide the benefits it is supposed to provide. If benefits are expected but not obtained, that too is a kind of harm.

Some studies describe the absence of anticipated health benefits as a safety issue, but it makes sense to distinguish the two as different types of quality issues. Failure of infants who use a particular type of infant formula to achieve anticipated weight gains, for example, usually would be regarded as a failure to achieve an anticipated health benefit, not as a safety issue. Similarly, overweight usually is not viewed as a food safety issue.

The tendency toward being overweight often begins in childhood [35]. As many people’s first processed food, infant formula might be a significant factor leading to overweight in childhood and throughout the lifespan [36,37]. Studies on being overweight have given little attention to its possible relationship to infant feeding methods.

Worldwide, there are many regulations relating to food safety, but foods are not regulated in relation to their effectiveness. Their producers generally do not make explicit claims regarding their effectiveness, and proof of effectiveness is not required before they are marketed. This is understandable because most foods are components in diverse diets, and it is difficult to isolate the benefits associated with any single type of food. Unfortunately, governments have taken this approach to the regulation of infant formula, so the manufacturers are not required to demonstrate that infant formula is nutritionally adequate in the sense of ensuring intellectual development, vision, and immune system development comparable to that obtained with breastfeeding [38].

The differences in impacts of different feeding methods should be assessed on many different dimensions. One breastfeeding advocacy group documents *21 Dangers of Infant Formula* [29]. Apart from the usual health oriented concerns listed there, feeding methods also affect children’s development on other dimensions, such as their lifelong immune functions and their visual acuity. Further, the choice of feeding methods also has implications relating to economic costs, environmental impact, convenience, and the mother’s self-image. There are many considerations that must be taken into account if we are to understand the choices parents make regarding how they feed their children.

A distinction should be made between impacts that are readily detected and those that can only be estimated based on statistical analyses of population data. One study estimated, “If 90% of US families could comply with medical recommendations to breastfeed exclusively for 6 months, the United States would . . . prevent an excess 911 deaths, nearly all of which would be in infants. . . [39] p. e1048” When this estimate was published it got little attention in U.S. or world media. In contrast, when the deliberate contamination of infant formula with melamine was reported to have led to six infant deaths in China, it aroused huge alarm in China and the world. The alarm has continued on for years, even though that particular cause of infant death is no longer in place.

Nutritional adequacy can be estimated only on the basis of population-based studies, conducted over time by expert researchers. Parents need to be informed of the scientific findings in ways that are fair and meaningful for them.

It is especially difficult to assess the impacts on child health of *shifts* from lower to higher levels of income. Overall, there is a clear trend of improvements in child health as families and countries move to higher income levels. However, increasing use of infant formula as incomes increase in emerging economies is likely to mean that the improvement in health associated with increased wealth is lower than it would have been if these families had instead practiced optimum breastfeeding. In terms of population-level trends, the positive health impact of the increase in wealth might mask the harm to health due to the shift from breastfeeding to infant formula.

Infant formula is regulated under weak national rules in some countries and not at all in other countries. One way to change that would be to have national law treat infant formula as a pharmaceutical, and not just as a food. This would be important because pharmaceuticals are assessed and regulated not only in terms of their safety but also their effectiveness. For infant formula, effectiveness would be equivalent to its nutritional adequacy.

Section 2.1.1 of the standards set out by the Codex Alimentarius Commission in 1981 said:

Infant formula means a breast-milk substitute specially manufactured to satisfy, by itself, the nutritional requirements of infants during the first months of life up to the introduction of appropriate complementary feeding [40] p. 1.

This means that infant formula should be viewed as nutritionally adequate only if it is proven to be as good for children as breastfeeding.

Most manufacturers do not claim that feeding with infant formula is as good as or better than breastfeeding for infants’ health. However, they do not provide the users of the product with systematic information about its nutritional inadequacies. Some parents believe infant formula is as good as or better than breastfeeding. Some reasonably objective agency should provide the information that is needed so that not only parents but also health workers and governments can make well-informed decisions about how to feed infants and young children.

Reclassifying infant formula as a pharmaceutical might not be politically feasible, but even so it would be interesting to know how it would be assessed if it were viewed as a pharmaceutical rather than as an ordinary food. Guidance for conducting such an analysis is available from the United States’ Institute of Medicine [41].

In the United States and some other countries, people who purchase pharmaceuticals must be told about the risks and side effects that might be involved in using them. What would be said if infant formula were to be categorized as a pharmaceutical? Whether or not the legal system treats infant formula as a pharmaceutical, it is important for parents, health workers, and governments to know not only about the safety but also the nutritional adequacy of the product.

The situation in China illustrates the importance of being attentive to the issue of nutritional adequacy. There are still strong feelings in the country about the infant formula contamination episode of 2008. As a result, the government has issued strong regulations to protect itself from similar scandals in the future [42,43]. Unfortunately, these regulations focus on food safety and fail to question the nutritional adequacy of the product. Apparently there is an unexamined assumption that if infant formula is safe, it will be good for the infants. This assumption places infants in China and everywhere else at risk. China and other countries that anticipate large increases in the use of infant formula among their people should consider monitoring the long-term health impact of these increases.

Some government agencies give useful advice on how to prepare and use infant formula safely [44]. Several promote breastfeeding in various ways. However, I have not been able to find any agency that provides systematic evidence-based information to guide parents’ choice between breastfeeding and feeding with formula. While there is much discussion about safety, there is practically no discussion about nutritional adequacy. There is a clear need for better management of the information needed by both parents and governments regarding the choice of infant feeding methods.

## Feeding alternatives

There is a basic recipe for infant formula recommended by the Codex Alimentarius Commission, the global organization that recommends standards for food products. However, there have been many variations in the product over time as various ingredients have been added or modified. The health claims for any specific type of infant formula usually are based on comparisons with other types of infant formula. That is not explained clearly, so some people may think the comparison is with breast milk.

The most important comparisons relating to infants’ health are between feeding with infant formula and feeding with breast milk, since the choice between those options is likely to make the biggest difference. The differences in health impacts between various versions of infant formula are likely to be small when compared with the differences between feeding with infant formula and breastfeeding.

It is generally agreed that the gold standard for infant feeding is breastfeeding, or more precisely, optimum breastfeeding. Breastfeeding is the norm against which other feeding methods should be compared. On that basis one should not speak of the advantages of breastfeeding, but of the harms associated with other methods of feeding [45,46].

The fundamental choice faced by most parents in high-income countries is between feeding with breast milk and feeding with infant formula, but that oversimplifies the issue. Breast milk can be provided in various ways. Mothers can breastfeed in ways that are optimal or sub-optimal. Breast milk can be expressed and placed into bottles or other devices and may be delivered by other people. There is also the possibility of providing breast milk obtained from women other than the biological mother. Breast milk from other women can be delivered through direct breastfeeding by wet nurses or by other people through bottles.

Infant formula can be delivered to the infant in various ways. Some mothers prop the bottle up on a pillow while attending to other matters. Some put their infants to bed with bottles. Some feed their infants whenever they cry. Some always encourage their infants to finish the bottle, which may increase the likelihood of becoming overweight [47].

Infant formula can be purchased in liquid or powdered forms. It can also be purchased in fast dissolving cubes. In Japan, the cubes accounts for 25 percent of infant formula sales [48].

There are choices of what to feed the infant beyond breastfeeding and commercial infant formula — not always wise choices. Some mothers produce their own home-made infant formula. Some feed their infants with goat’s milk or cow’s milk, perhaps with a bit of sugar added. Some women feed their infants tea or cola.

Many different kinds of formula are available. The focus here is on formula for infants up to about six months of age. But the issues highlighted here are related to those for formula designed for older children or children with special concerns such as overweight [49]. One of the linkages is through the manufacturers’ interest in establishing brand loyalty. The manufacturers press families to start with their brand of formula as early as possible, and stay with them, not only for “toddler formula” but also for other forms of baby food.

It is usually assumed that any reference to commercial infant formula refers to the product based on cow’s milk. However, some manufacturers are promoting infant formula based on goat-milk [50]. Soy-based infant formula is a well-established commercial alternative, especially for lactose intolerant infants.

Assessments of the quality of food products involve comparisons. For *safety* issues, typically the comparison is in terms of health outcomes following consumption of the food product with and without particular contaminants or with and without particular types of mishandling of the product. Infant formula can be mishandled by, say, ignoring use-by dates, failing to wash the utensils properly, or mixing the powder with unclean water.

In assessing health *benefits* of any particular food, the comparison is with other food, or with the same food in a modified form. For example, one might compare the health benefits from using a particular type of infant formula with the health benefits from using the same formula but with a specific additive.

Basic commercial infant formula itself has changed over time:

In the first decades of the 20th century, most non-breastfed infants received formulations based on whole cow’s milk or top milk, with high sodium concentrations and levels of cholesterol and fatty acids that were similar to those in mature breast milk. By the 1950s, commercially prepared formulas became increasingly popular. At this time, formulas tended to have high sodium concentrations and low levels of iron and essential fatty acids. Starting in the 1980s, sodium content was reduced and nowadays the majority of formulas have levels similar to those in breast milk [51] p. 5.

Over time, the manufacturers have claimed various improvements in infant formula with respect to its safety and its list of ingredients. However, it is not clear whether these improvements have led to significantly better health outcomes for infants. The health impacts remain questionable because few studies have been done on them. To illustrate, while many people claim that fatty acid additives to infant formula would increase children’s intelligence, the evidence to support that claim is weak. There have been no field studies comparing the intelligence of children before and after the manufacturers began including that additive into infant formula [52].

For cases in which mothers do not breastfeed their own children, there is increasing interest in the sharing of breast milk, especially in high-income countries [53-55]. In some countries, wet nurses are employed. Many parents now recognize that commercial infant formula is not the only alternative to breastfeeding by the infant’s biological mother.

Some studies of the impacts of breastfeeding fail to specify the alternative: Commercial formula? Which commercial formula? Home-made formula? Based on what recipe? Cow’s milk? Goat’s milk? Tea? Delivered how? Studies of child feeding should focus on the *difference* in the health impacts of two specific methods of feeding, both of which should be described. If the alternatives are not specified, the meaning of the research findings would be clouded.

This is especially important when making comparisons across countries. As mentioned earlier, one study estimated that proper breastfeeding could prevent 911 deaths per year in the U.S [39]. Another study said that in India 800,000 infants die each year as a result of sub-optimal breastfeeding [56]. India’s mortality numbers are larger than those in the U.S. not only because the country’s population is much larger, but also because the infant formula and other breast milk substitutes used in India are less likely to be handled safely. These mortality estimates are based on comparisons of optimum breastfeeding with different existing feeding patterns. There are distinct differences: “In high-income countries, the babies usually receive industrialized formula, whereas many non-breastfed infants in the low and middle income countries receive whole or diluted animal milk [51] p. 6.”

In many cases, infants are fed with some breast milk and some infant formula, or something else. This makes it difficult to make clean comparisons between the effects of feeding with infant formula and breastfeeding. Some studies code infants simply as predominantly formula-fed or predominantly breastfed. This will show smaller differences than would have been obtained by comparing exclusive breastfeeding and exclusive formula feeding. If no distinction is made between infants whose diet is comprised of one percent infant formula and those whose diets are comprised of ninety-nine percent infant formula, the research findings will be muddled.

There is little large-scale published research that compares the health impacts of breastfeeding with the health impacts of feeding with formula. There would be methodological difficulties in doing such research, but the main impediment is the lack of funding for it. With the intense promotion of infant formula that is now underway, consideration should be given to going beyond short-term localized studies to also launch sustained monitoring of the long-term health impacts of changing patterns of infant feeding. This monitoring could be carried out through modifications of record-keeping practices in health care facilities that track children over time.

## Global guidance

Clear consensus-based guidance with respect to the feeding of infants and young children has already been established at the global level. In the *Global Strategy for Infant and Young Child Feeding* the World Health Organization and the United Nations Children’s Fund describe the basis for the strategy as follows:

Breastfeeding is an unequalled way of providing ideal food for the healthy growth and development of infants; it is also an integral part of the reproductive process with important implications for the health of mothers. As a global public health recommendation, infants should be exclusively breastfed for the first six months of life to achieve optimal growth, development and health. Thereafter, to meet t heir evolving nutritional requirements, infants should receive nutritionally adequate and safe complementary foods while breastfeeding continues for up to two years of age or beyond [57] pp. 7–8.

At the global level, the Codex Alimentarius Commission develops non-binding guidelines regarding food composition and safety, including infant formula. In 1976, at its 11th session, the Commission issued a *Statement on Infant Feeding* that said, “it is necessary to encourage breastfeeding by all possible means in order to prevent that the decline in breastfeeding, which seems to be actually occurring, does not lead to artificial methods of infant feeding which could be inadequate or could have an adverse effect on the health of the infant [58] p. 1.”

At this session the Commission also adopted a *Codex Standard for Infant Formula*. Designated as CODEX STAN 72–1981. The standard includes a list of required ingredients and names various required quality control measures. Several amendments have been adopted since then, but this core statement of the required ingredients for infant formula is still accepted throughout the world. The standards are widely regarded as a minimum standard, thus allowing the marketing of a broad variety of infant formulas.

Widespread concern over the ways in which infant formula has been promoted led to the adoption of the *International Code of Marketing of Breast-milk Substitutes* by the World Health Assembly in 1981 [59]. Subsequent resolutions of the World Health Assembly help to clarify and extend the Code. The International Baby Food Action Network, a strong global network of non-governmental organizations, helps to ensure the implementation of the Code, It issues regular reports on actions taken by national governments to implement the Code [4]. The World Health Organization also has reported on this issue [60]. Many countries have incorporated some or all of the Code into their national law and created national government agencies to oversee its implementation.

Instead of regulation by the government, some countries rely on some form of self-regulation by the industry. In Australia, for example, there has been a *Manufacturers and Importers Agreement* since 1992, “a voluntary self-regulatory code of conduct between the manufacturers and importers of infant formula in Australia [61] p. 1.” Many observers were disappointed when the Advisory Panel on the Marketing in Australia of Infant Formula ceased to operate in November 2013. It was supposed to monitor industry compliance with the agreement. That function is now handled through a standardized complaint form to be submitted to the Department of Health.

The global instruments offer recommendations, not binding law. Some countries have specific binding regulations relating to infant formula in their national law, but many do not. Most accept the Codex Alimentarius recommendations. Together, the Codex recommendations and the Code constitute the current global governance framework for infant formula.

Some people might have thought that with the adoption of the Code the need for regulation at the global level was met. However, the Code needs to be strengthened in several ways:

1. It needs to be made clear that the Code applies to all countries, not just low-income countries.
2. The Code needs to be updated to recognize that some governments, and not just companies, promote the use of infant formula in ways that are contrary to the principles set out in the Code.
3. There is a need to clarify and strengthen the application of the Code in international trade and other international relations.
4. The Code is sometimes viewed as applying only to infant formula, so its applicability to other breast-milk substitutes needs to be clarified.
5. The Code should be adapted and placed into the international human rights framework [62] pp. 103–106.

The Code focuses on the ways in which infant formula is marketed. There are other issues of concern. There is a need to update the basic recipe for infant formula. There is a need to ensure that quality standards are uniform across the globe. There is a need to constantly improve both the safety and the nutritional adequacy of infant formula. There is a need to promote awareness of good alternatives to direct feeding by the biological mother, such as the use of donor milk under well-controlled conditions. There is a need to improve the quality of information that is available to parents, health workers, and governments regarding the question of how infants and young children should be fed.

The baby food industry is being globalized at an unprecedented pace, amplifying concerns of the sort described here. While other elements of food systems might be controlled locally, the baby food industry needs some form of global governance to ensure that infants and young children everywhere are well nourished.

## Prospects for global regulation

The potential for regulation of the baby food industry from the global level is limited, but it is possible to make improvements over the current situation. There is a need to respect the sovereignty of the nation-states. There is no global government, but there is global *governance*. Nations can, by consensus, agree to adopt specific rules and guidelines, expressed in documents of various forms.

In principle, treaties are binding on those states that commit to support them through their national ratification processes. They are then obligated to conform their national laws to support those commitments.

Other forms of agreement are less binding, but nevertheless contribute to orderly global governance. Some of them offer strong recommendations formulated through consensus. It is through these mechanisms that we have the possibility of orderly global management of services in the public interest. The management of international air traffic provides a good example of this sort of global governance.

Regulation from the global level must be based on the articulation of clearly negotiated consensus among the nations of the global community. The function of this sort of regulation is not to set out detailed and binding requirements on those nations, but to set out basic principles to be followed and implemented by national governments through their national laws.

The concept of the human right to adequate food has a long history, but it came to maturity in the 1990s, driven by nongovernmental groups working with the United Nations system [63-66]. Good progress could be made on the issues discussed here through a focus on the human right to adequate food specifically as it applies to children.

These rights need to be clearly articulated. This could be done through a new Optional Protocol to the Convention on the Rights of the Child that focuses on children’s nutrition. Other possibilities would be a new General Comment on the topic, or new right to food guidelines that focus specifically on infants and young children. Any of these could be produced through well-established procedures of the United Nations system, under the guidance of the Office of the High Commissioner for Human Rights, the Food and Agriculture Organization of the United Nations, the World Health Organization, and the United Nations Children’s Fund.

That Convention on the Rights of the Child already has two Optional Protocols associated with it, one on the involvement of children in armed conflict, and another on the sale of children, child prostitution, and child pornography. Their forms could suggest a structure for a new Optional Protocol on Children’s Nutrition (OPCN).

Working under the auspices of the United Nations General Assembly, the nations of the world could negotiate a draft OPCN. Drafts could be prepared by national governments working together with non-governmental organizations. The drafters could draw from the many documents that already propose sound principles relating to children’s nutrition such as the World Health Organization’s *Global Strategy for Infant and Young Child Feeding* and the *International Code of Marketing of Breast-milk Substitutes*. There are many other relevant documents, now scattered, whose core ideas could be pulled together.

When a draft for the OPCN was ready, the General Assembly of the United Nations would vote on it. If a majority agreed, it would be adopted by the UN’s General Assembly.

From that point forward, the executive branches of the national governments of the world would be invited to sign the OPCN, and then have their national legislatures or other appropriate bodies ratify it, in the normal procedure used to signify nations’ agreements to international treaties.

Ratification would indicate the nation’s acceptance of the OPCN and its commitment to conform its national laws to it. Following ratification, the broad principles stated in the OPCN would be given concrete form through the adoption of appropriate national laws. The ratification would signify the nation’s willingness to be held accountable with regard to the principles stated in the OPCN, and the new national laws would be the means by which its leaders would act on its commitment.

The OPCN would not replace international bodies such as UNICEF or the Codex Alimentarius Commission, nor would it replace national regulatory agencies such as the Food and Drug Administration in the U.S. The OPCN would help to harmonize the work of all participating countries at the national level. It would be the apex document, setting out important principles relating to the nutrition of infants and young children.

The drafters of the OPCN would have to accommodate diversity and recognize the important differences in cultural approaches to raising children in different places [67]. As a global document, it would focus mainly on widely accepted principles, and leave the details of implementation to be worked out in different countries according to their particular circumstances.

National regulatory and operational bodies, while functioning independently, would be free to obtain guidance from relevant global agencies as the Food and Agriculture Organization of the United Nations, the United Nations Children’s Fund, and the World Health Organization.

In time, another document could be prepared to suggest concrete ways in which national governments could implement the principles of the OPCN, comparable to the *Voluntary Guidelines to Support the Progressive Realization of the Right to Adequate Food in the Context of National Food Security* [68]. A document of this sort could, for example, set out guidelines for field research to compare the qualities of alternative methods of child feeding.

This approach would place children’s nutrition decisively into the human rights framework. Like other forms of international law, it would not result in immediate compliance, but it would establish clear and widely agreed standards, and it would support the formation of strong law at the national level. A new Optional Protocol on Children’s Nutrition, linked to the Convention on the Rights of the Child, would help to establish coherent regulations for ensuring that infants and young children everywhere are well nourished.

## References

[1] Richter J (2001). Holding Corporations Accountable: Corporate Conduct, International Codes, and Citizen Action.

[2] GRAIN. The great milk robbery: How corporations are stealing livelihoods and a vital source of nutrition from the poor*.* GRAIN. December 7, 2011. http://www.grain.org/article/entries/4259-the-great-milk-robbery-how-corporations-are-stealing-livelihoods-and-a-vital-source-of-nutrition-from-the-poor

[3] Gomes A (2011). Local milk within Hawaii’s grasp. Honolulu Star-Advertiser.

[4] IBFAN International Baby Food Action Network. Website. http://www.ibfan.org/

[5] Kimura AH (2013). Hidden Hunger: Gender and the Politics of Smarter Foods.

[6] Shetty P (2014). Indonesia’s breastfeeding challenge is echoed the world over. Bull World Health Organ.

[7] Transparency Market Research (2013). Asia Pacific Baby Food Market—Global Industry Analysis, Size, Share, Growth, Trends and Forecast, 2013–2019.

[8] UBIC Consulting (2014). Ingredients for the World Infant Formula Market.

[9] Trade Arabia (2014). Mena baby food market booming. Trade Arabia Business News Information.

[10] Bandy L (2014). Toddler milk formula: The Hello Kitty of packaged food. Euromonitor Int.

[11] Adams C (2014). Fonterra Spreads Infant Formula Sales in China.

[12] Astley M (2014). Nestlé to build $350m infant formula plant in Mexico. DairyReportercom.

[13] Bender R (2014). France Has a formula for China’s baby-milk needs. Wall Street J.

[14] The Economist (2014). Spilt milk in China. ᅟ.

[15] Montague Jones G (2010). China uncovers more melamine tainted milk powder. Food Prod Dailycom.

[16] Yan J (2011). Fonterra in the San Lu Milk Scandal—a Case Study of a New Zealand Company in a Product-harm Crisis.

[17] Chibber A (2014). Baby food boom in China is emptying Australian supermarket shelves. Dairy Reportercom.

[18] Adams C (2014). End of the white goldrush?. New Zealand Herald.

[19] Kitney D (2014). United dairy power boss targets massive Chinese market. Aust.

[20] Wyatt D (2013). Ai Weiwei unveils milk tin map of China in new protest piece against baby formula scandal. Independent (United Kingdom).

[21] Vega PC. Art review: Ai Weiwei’s ‘Baby Formula’: A case for consuming art. March 12, 2014. GMA News Online. http://www.gmanetwork.com/news/story/352204/lifestyle/artandculture/art-review-ai-weiwei-s-baby-formula-a-case-for-consuming-art

[22] Coriolis (2014). Infant Formula Value Chain.

[23] Euromonitor International. Website. http://www.euromonitor.com/baby-food

[24] UNICEF (2013). Breastfeeding on the Worldwide Agenda.

[25] Baumslag N (2006). Mother & Child Health—Common Sense, Creativity and Care. Selected Works of Dr. Cicely D. Williams, Primary Health Care Pioneer.

[26] Krasny J (2012). Every parent should know the scandalous history of infant formula. Bus Insider.

[27] Muller M (1974). The Baby Killer.

[28] Ip S, Chung M, Raman G, Chew P, Magula N, DeVine D, Trikalinos T, Lau J (2007). Breastfeeding and Maternal and Infant Health Outcomes in Developed Countries.

[29] WABA (2012). 21 Dangers of Infant Formula.

[30] Wall G (2013). Outcomes of Breastfeeding.

[31] World Health Organization (2007). Evidence on the Long-Term Effects of Breastfeeding.

[32] World Health Organization (2013). Short-Term Effects of Breastfeeding: A Systematic Review on the Benefits of Breastfeeding on Diarrhea and Pneumonia Mortality.

[33] Fitzsimons E, Vera Hernandez M (2013). Food for Thought? Breastfeeding and Child Development.

[34] Nutt D, King LA, Saulsbury W, Blakemore C (2007). Development of a rational scale to assess the harm of drugs of potential misuse. Lancet.

[35] Cunningham SA, Kramer MR, Venkat Narayan KM (2014). Incidence of childhood obesity in the United States. N Engl J Med.

[36] Rose DJ, Bodor N, Chilton M (2006). Has the WIC incentive to formula-feed led to an increase in overweight children?. J Nutr.

[37] WIC (2006). Breastfeeding: The First Defense Against Obesity.

[38] Kent G (2012). The nutritional adequacy of infant formula. Clin Lact.

[39] Bartick M, Reinhold A (2010). The burden of suboptimal breastfeeding in the United States: a pediatric cost analysis. Pediatrics.

[40] Codex Alimentarius Commission. Standard for Infant Formula and Formulas for Special Medical Purposes Intended for Infants. CODEX STAN 72–108. [Formerly CAC/RS 72–1972. Adopted as a world-wide Standard 1981.Amended 1983, 1985,1987. Revision 2007.] http://www.codexalimentarius.net/download/standards/288/CXS_072e.pdf

[41] Institute of Medicine (2012). Safe and Effective Medicines for Children: Pediatric Studies Conducted Under the BPCA and PREA.

[42] Burkitt L (2013). China to strengthen infant formula regulations. Wall Street J.

[43] Xinhua (2013). China Focus: China Unveils Tighter Regulation on Infant Formula Producers.

[44] Government of South Australia (2014). Infant Formula: A Guide to Safe Preparation and Feeding of Infant Formula.

[45] McNiel M, Labbok M, Abrahams SW (2010). What are the risks associated with formula feeding? A re-analysis and review. Birth.

[46] Wiessinger D (1996). Watch your language!. J Hum Lact.

[47] Perrin EM, Rothman RL, Sanders LM, Skinner AC, Eden SK, Shintani A, Throop EM, Yin S (2014). Racial and ethnic differences associated with feeding- and activity-related behaviors in infants. Pediatrics.

[48] Astley M (2014). Asia would be ‘nice next step’ for infant formula cube: Meiji. DairyReportercom.

[49] Poulter S (2013). Mums ‘waste £500 a year’ on toddler formula: Ordinary cow’s milk is healthier and cheaper, says Which?. Mail Online.

[50] Research and Markets (2014). China Goat Milk Infant Formula Industry Report 2014.

[51] Horta BL, Victora CG (2013). Long-Term Effects of Breastfeeding: A Systematic Review.

[52] Kent G (2014). Regulating fatty acids in infant formula: critical assessment of U.S. policies and practices. Int Breastfeed J.

[53] Akre JE, Gribble KD, Minchin M (2011). Milk sharing: from private practice to public pursuit. Int Breastfeed J.

[54] Huff EA (2012). Popularity of mothers’ milk banks soars as more women ditch infant formula. Nat News.

[55] Tigchelaar P, Goodell S, Jonathan CA, Fogleman A (2014). A mixed-methods observational study of human milk sharing communities on Facebook. Breastfeed Med.

[56] Press Trust of India (2013). 8 lakh [800,000] infants die every year due to inadequate breastfeeding. ᅟ.

[57] World Health Organization (2003). Global Strategy for Infant and Young Child Feeding.

[58] Codex Alimentarius Commission. Codex Alimentarius Commission. Statement on Infant Feeding, CAC/MISC-2-1976. 1976. http://www.codexalimentarius.net/download/standards/301/CXA_002e.pdf

[59] World Health Organization (1981). International Code of Marketing of Breast-Milk Substitutes.

[60] World Health Organization (2011). Country Implementation of the International Code of Marketing of Breast-Milk Substitutes: Status Report 2011.

[61] Australian Government. Department of Health (2014). Marketing in Australia of Infant Formulas: Manufacturers and Importers Agreement.

[62] Kent G (2011). Regulating Infant Formula.

[63] Kent G (2004). Human rights and infant nutrition. WABA Global Forum II-23-27 September 2002-Arusha, Tanzania.

[64] Kent G (2005). Freedom from Want: The Human Right to Adequate Food.

[65] Kent G (2006). Child feeding and human rights. Int Breastfeed J.

[66] Kent G (2008). Global Obligations for the Right to Food.

[67] DeLoache J, Gottlieb A (2000). A World of Babies: Imagined Childcare Guides for Seven Societies.

[68] Food and Agriculture Organization of the United Nations (2005). Voluntary Guidelines to Support the Progressive Realization of the Right to Adequate Food in the Context of National Food Security.

